# Co-Reactivation of Cytomegalovirus and Epstein-Barr Virus Was Associated With Poor Prognosis After Allogeneic Stem Cell Transplantation

**DOI:** 10.3389/fimmu.2020.620891

**Published:** 2021-02-16

**Authors:** Jing-Rui Zhou, Da-Yu Shi, Rong Wei, Yu Wang, Chen-Hua Yan, Xiao-Hui Zhang, Lan-Ping Xu, Kai-Yan Liu, Xiao-Jun Huang, Yu-Qian Sun

**Affiliations:** ^1^ Peking University People's Hospital, Peking University Institute of Hematology, National Clinical Research Center for Treatment of Hematological Disease, Beijing, China; ^2^ Beijing Key Laboratory of Hematopoietic Stem Cell Transplantation for the Treatment of Hematological Diseases, Beijing, China; ^3^ Peking-Tsinghua Center for Life Sciences, Beijing, China

**Keywords:** cytomegalovirus, Epstein-Barr virus, co-reactivation, immune reconstitution, stem cell transplantation

## Abstract

Reactivation of cytomegalovirus (CMV) or Epstein-Barr virus (EBV) is common after hematopoietic stem cell transplantation (HSCT). Previous researches have demonstrated that either CMV or EBV reactivation is associated with poor outcomes of HSCT. However, few studies investigate the impact of CMV and EBV co-reactivation after HSCT. In this study, we described the clinical characteristics of HSCT recipients with CMV and EBV co-reactivation (defined as CMV and EBV viremia occur at the same period of time). We conducted a longitudinal study of 247 patients who underwent HSCT in our center. A total of 24 (9.7%) patients had CMV and EBV co-reactivation. These patients showed higher incidence of viral pneumonitis (P=0.005). Patients with CMV and EBV co-reactivation had significant lower 1-year overall survival (OS) (P=0.004) and lower 1-year leukemia free survival (LFS) (P=0.016). Our further analysis suggested that duration of CMV (P=0.014), EBV (P<0.001), and CD4+CD25+ T cell counts at day 30 post-transplantation (P=0.05) are independent risk factors of virus co-reactivation. In conclusion, patients who developed co-reactivation of CMV and EBV had poor prognosis in terms of lower 1-year OS and LFS, and the CMV and EBV co-reactivation was associated with prolonged CMV or EBV duration and poor CD4+CD25+ T cell reconstitution at day 30 post-transplantation.

## Introduction

The burden of clinically relevant viral infections, especially double-stranded DNA herpesviruses, continues to rise. Reactivation of multiple different herpes viruses is commonly acquired following allogeneic hematopoietic stem cell transplantation (HSCT). Cytomegalovirus (CMV) is the most frequently reactivated virus (1) after allo-HSCT and increases non-relapse mortality despite the widely adopted protocol of pre-emptive therapy (2–4). Epstein-Barr virus (EBV) (1), especially EBV-related post-transplantation lymphoproliferative disorder (PTLD), is associated with a high mortality rate of 50%–90% (5, 6).

CMV and EBV are the most clinically relevant viruses in the present era with well-defined treatment approaches. A bidirectional relationship seems to exist between these two viruses; higher incidence/poor clearance of CMV infection and a higher incidence of EBV-PTLD and delayed immune reconstitution as a cause or effect is key to all these findings (7, 8). It is therefore reasonable to assume that co-reactivation of CMV and EBV may indicate an even more severe clinical condition compared to that for the reactivation of each virus alone. However, few studies have investigated co-reactivation of CMV and EBV among HSCT recipients. In our study, we aimed to explore the clinical characteristics of patients with co-reactivation of CMV and EBV, study the effect of such co-reactivation on prognosis, and identify associated risk factors. We also discuss the role of immune reconstitution in the co-reactivation of the two viruses.

## Materials and Methods

### Study Cohort

A total of 253 patients underwent their first allo-HSCT between July 2015 and June 2016 at Peking University People’s Hospital (Haidian district, Beijing) at the Institute of Hematology. These patients were retrospectively reviewed in the current study. The Ethics Committee of Peking University People’s Hospital approved this study. All patients provided written informed consent prior to transplantation.

### Transplantation Procedure

For patients with acute leukemia (AL) or myelodysplastic syndrome (MDS) who underwent haplo-HSCT and matched unrelated donor HSCT, the conditioning regimen consisted of cytarabine (4 g/m^2^/day) intravenously on days -10 to -9, busulfan (3.2 mg/kg/day) intravenously on days -8 to -6, cyclophosphamide (1.8 g/m^2^/day) intravenously on days -5 to -4, semustine (250 mg/m^2^) orally once on day -3, and rabbit anti-thymocyte globulin (ATG) (2.5 mg/kg/day; Sang Stat, Lyon, France) intravenously on days -5 to -2. Patients with AL or MDS who underwent HLA-identical HSCT received a conditioning regimen that did not include ATG but consisted of hydroxyurea (80 mg/kg) orally divided twice on day -10, cytarabine (2 g/m^2^/day) intravenously on day -9, busulfan (3.2 mg/kg/day) intravenously on days -8 to -6, cyclophosphamide (1.8 g/m^2^/day) intravenously on days -5 to -4, and semustine (250 mg/m^2^) orally once on day -3. For patients with aplastic anemia who underwent haplo-HSCT, conditioning therapy consisted of busulfan (3.2 mg/kg/day) intravenously for 2 days on days -7 and -6, cyclophosphamide (50 mg/kg/day) intravenously for four consecutive days on days -5 to -2, and rabbit ATG (2.5 mg/kg/day; Sang Stat, Lyon, France) intravenously for four consecutive days on days -5 to -2 (9). For patients with aplastic anemia who underwent identical HSCT or matched unrelated donor HSCT, the conditioning regimen excluded busulfan, and only consisted of cyclophosphamide (50 mg/kg/day) intravenously for four consecutive days on days -5 to -2, and rabbit ATG (2.5 mg/kg/day; Sang Stat, Lyon, France) intravenously for four consecutive days on days -5 to -2. The conditioning regime of the only one MM patient in this study consisted of cytarabine (4 g/m^2^/day) on days -10 to -9, busulfan (3.2 mg/kg/day) on days -10 to -8, cyclophosphamide (1g/m^2^/day) on days -7 to -6, fludarabine 50 mg/day on days -6 to -2, and simustine (250 mg/m^2^) orally once on day -3 along with rabbit ATG (2.5 mg/kg/day) on days -5 to -2.

### Virus Monitoring and Therapy

CMV and EBV reactivation was monitored twice per week using real-time quantitative polymerase chain reaction (PCR) of plasma samples. All patients received ganciclovir between days -9 and -2 (10). Pre-emptive therapy with either intravenous ganciclovir (5 mg/kg, twice daily) or intravenous foscarnet (90 mg/kg/d) was initiated when CMV viremia was confirmed and the treatment lasted until CMV DNA was not detected twice on consecutive tests. Adoptive transfer of CMV-specific cytotoxic T lymphocytes (CTLs) was performed if available in those with refractory CMV infection or CMV disease (2). Antiviral drugs, such as foscarnet, were infused in patients with EBV viremia. In addition, rituximab was infused if EBV viremia was persistent or developed into EBV disease (6). EBV-specific CTL therapy was adopted as salvage option.

### Graft-Versus-Host Disease (GVHD) Prophylaxis

Cyclosporin A (CsA), methotrexate (MTX), and mycophenolate (MMF) were administered to patients for GVHD prophylaxis. CsA was administered at 2.5 mg/kg/day intravenously in two doses from day -9 until the patients could take CsA orally. The trough concentration of CsA was monitored, requiring a target trough blood concentration of 150–250 ng/ml. MTX was administered intravenously at a dose of 15 mg/m^2^ on day +1 and 10 mg/m^2^ on days +3, +6, and +11 (day +11 was omitted in patients with matched sibling donor transplantation). Mycophenolate (MMF) was administered orally from day -9 to day +30 at a dose of 0.5 g (0.25 g for children) every 12 h.

### Immunophenotyping

Peripheral blood samples were collected from recipients on days 30, 60, and 90 after HSCT. The samples were stained without further separation to minimize selective loss shortly after collection. The combinations of the directly conjugated monoclonal antibodies CD3-FITC, CD4-PE, CD8-APC, CD19-Per-CP, CD25-PE (BD Biosciences, Mountain View, CA, USA), and their isotype-matched antibodies were used to analyze the immunophenotype of T lymphocyte subsets. Flow cytometry was performed using a BD FACSSort machine (Becton Dickinson Biosciences, San Jose, CA, USA). The data were analyzed using CellQuest software (BD Biosciences).

### Definitions

Myeloid engraftment was defined as the first of three consecutive days with an absolute neutrophil count (ANC) ≥0.5×10^9^/L, and platelet engraftment was defined as the first of seven consecutive days with a platelet count ≥20×10^9^/L without transfusion. CMV and EBV viremia was defined as the first of two consecutive detections in which virus DNA reached or exceeded 1,000 copies/ml and 500 copies/ml, respectively. Co-reactivation of CMV and EBV was defined as the detection of EBV or CMV viremia during CMV or EBV viremia, respectively. The time of co-reactivation was defined as the day when viremia of the first virus was identified. The duration of viremia was defined as the number of days between the first day of viremia and the first day when the virus was no longer found. The longest duration was included in the analysis of patients with more than one episode of viremia. CMV disease was diagnosed according to the published definition. Both acute and chronic GVHD were diagnosed and graded using traditional criteria (11, 12). Time to relapse was defined as days between date of transplantation and date of disease recurrence. Non-relapse mortality (NRM) was defined as death from all causes other than those directly related to a hematologic malignant disease itself, occurring at any time after transplantation. Overall survival (OS) was defined as the number of days from transplantation to death from any cause. Leukemia-free survival (LFS) was defined as the number of days from transplantation to disease progression after transplantation.

### Statistical Analyses

Categorical variables were compared between the two groups using the χ^2^ test or Fisher’s exact test. Continuous variables were compared using a nonparametric test (Mann-Whitney U test). Multivariate Cox proportional hazards models were adopted with proportional hazards assumption and for testing interactions. Statistical analyses were performed using IBM SPSS 22.0 statistical software (IBM SPSS Statistics, USA).

## Results

### Patients Characteristics

Six patients infected with EBV were excluded from the study. Finally, 247 patients were enrolled in this study. Patient characteristics are listed in **Table 1**. There were 144 (58.3%) men. The median age was 29 (1–63) years. Acute leukemia, both acute myeloid leukemia (n=100, 40.5%) and acute lymphoblastic leukemia (n=107, 43.3%), accounted for most patients. More than half (n=194, 78.5%) of patients underwent HSCT from haploidentical donors. Forty-five (18.2%) patients received HSCT from HLA-matched siblings, and eight (3.2%) underwent HSCT from unrelated donors. Myeloid engraftment and platelet engraftment were achieved in 245 (99.2%) patients at a median of 13 (10–31) days and in 227 (91.9%) patients at a median of 14 (6–267) days after HSCT, respectively. The incidence of grade 3–4 acute GVHD and grade 1–4 acute GVHD was 6.12% (n=15) and 50.6% (n=125), respectively. The median follow-up time for survivors was 12 months. The 1-year OS, LFS, NRM, and relapse rates were 67.6%, 66.0%, 19.4%, and 6.5%, respectively.

**Table 1:** Characteristics of patients.

Characteristic	Co-reactivation group	Other reactivation group^#^	No reactivation group	*P* value
**Gender, no.(%)**				0.91
**Male**	14 (58.3)	83 (57.2)	47 (60.3)	
**Female**	10 (41.7)	62 (42.8)	31 (39.7)	
**Age, median (range)**	29 (6–51)	27 (1–61)	35 (3–63)	0.246
**Underlying disease, no.(%)**				0.22
** AML**	9 (37.5)	57 (39.3)	34 (43.6)	
** ALL**	14 (58.3)	66 (45.5)	27 (34.6)	
** SAA**	0	8 (5.5)	8 (10.3)	
** MDS**	1 (4.2)	8 (5.5)	8 (10.3)	
Other*	0	6 (4.1)	1 (1.3)	
**Disease status**				0.496
** ≤CR2**	23 (95.8)	133 (91.7)	69 (88.5)	
** CR3 or NR**	1 (4.2)	12 (8.3)	9 (11.5)	
**Donor-recipient relationship, no.(%)**				0.001
** Father**	13 (54.2)	66 (45.5)	20 (25.6)	
** Mother**	1 (4.2)	8 (5.5)	4 (5.1)	
** Sibling**	3 (12.5)	45 (31)	45 (57.7)	
** Son/Daughter**	5 (20.8)	23 (15.9)	6 (7.7)	
** Unrelated donor**	2 (8.3)	3 (2.1)	3 (3.8)	
**HLA match, no.(%)**				<0.001
** Haploidentical**	22 (91.7)	133 (91.7)	39 (50)	
** Identical**	0	9 (6.2)	36 (46.2)	
** Unrelated donor**	2 (8.3)	3 (2.1)	3 (3.8)	
**Blood type, no.(%)**				0.889
** Matched**	12 (50)	80 (55.5)	43 (55.1)	
** Minor mismatched**	4 (16.7)	29 (20)	13 (16.7)	
** Major mismatched**	5 (20.8)	28 (19.3)	15 (19.2)	
** Major and minor mismatched**	3 (12.5)	8 (5.5)	7 (9)	
**ATG used in conditioning therapy, no.(%)**	24 (100)	138 (95.2)	43 (55.1)	<0.001
**MNC, median (range), 10^8^/kg**	8.21 (5.91–13.35)	8.63 (4.3–15.67)	8.45 (2.89–12.74)	0.547
**CD34+ cell absolute count, median (range), 10^6^/kg**	3.14 (1.06–7.48)	2.41 (0.28–8.07)	2.49 (0.97–6.06)	0.409
**Donor gender, no.(%)**				0.148
** Male**	19 (79.2)	117 (80.7)	54 (69.2)	
** Female**	5 (20.8)	28 (19.3)	24 (30.8)	

^#^Other reactivation group includes reactivation of CMV only, and reactivation of both CMV and EBV but does not fulfill definition of CMV and EBV co-reactivation

*Other underlying diseases include multiple myeloma (one patient), chronic myelomonocytic leukemia (two patients), chronic myeloid leukemia (two patients), acute heterozygosis leukemia (two patients).

### Virus Reactivation

At least one episode of CMV viremia was found in 68.4% of the patients (n=169), among which 15 patients were infected twice or more during the year after transplantation. The median onset time of CMV viremia was 34 (7–175) days, and the median duration was 20 days (range, 6–77 days). CMV DNA copy numbers varied in patients with a median of 5.48×10^3^ (0–5.01 ×10^5^) copies. Thirty-six (14.6%) patients had EBV reactivation. EBV viremia occurred at a median of 48.5 (25–102) days after transplantation and lasted a median of 14 (3–60) days. For patients with reactivated EBV, EBV DNA copies reached 6×10^3^ (6×10^2^–1.76 ×10^6^). According to the definition above, 24 (9.7%) patients were categorized as having co-reactivation of CMV and EBV. Twelve (4.9%) patients had both CMV and EBV reactivation but did not fulfill the definition of co-reactivation. A total of 133 (53.8%) patients were infected with CMV only, and 78 (31.6%) patients had no episodes of reactivation of either virus.

### Effect of CMV and EBV Co-Reactivation on Clinical Outcomes

Patients were divided into three groups based on CMV and EBV reactivation according to our definition above: (1) co-reactivation group, defined as the detection of EBV or CMV viremia during CMV or EBV viremia, respectively; (2) other reactivation group was defined as reactivation with CMV and/or EBV but did not meet the criteria for co-reactivation; and (3) no reactivation group was defined as neither CMV nor EBV reactivation detected. The characteristics of the three groups are listed in **Table 1**.

Myeloid engraftment was comparable between the three groups (100% vs. 100% vs. 97.4% for co-reactivation, other reactivation, and no reactivation groups, respectively, *P*=0.113). However, myeloid engraftment seemed to be delayed in patients with no virus reactivation (13 vs. 13 vs. 14 days for co-reactivation, other reactivation, and no reactivation groups, respectively, *P*=0.008). Regarding platelet engraftment, the proportion of patients (87.5% vs. 91.7% vs. 93.6% for co-reactivation, other reactivation, and no reactivation groups, respectively, *P*=0.628) and days of engraftment (13 vs. 13 vs. 14 for co-reactivation, other reactivation, and no reactivation groups, respectively, *P*=0.389) were comparable between the three groups. The incidence of acute GVHD was significantly higher in the reactivation group than in the no reactivation group (50% vs. 66.9% vs. 20.5%, respectively, *P*<0.001), while the incidence of chronic GVHD was similar in the three groups (4.2% vs. 9.7% vs. 9% for co-reactivation, other reactivation, and no reactivation groups, respectively, *P*=0.682). Patients in the reactivation group were more likely to develop viral pneumonia than those in the other two groups (20.8% vs 9% vs 1.3% for co-reactivation, other reactivation, and no reactivation groups, respectively, *P*=0.005), but we did not observe a similar trend for viral enteritis (0% vs 2.1% vs. 0% for co-reactivation, other reactivation, and no reactivation groups, respectively, *P*=0.344). CMV or EBV disease was diagnosed in 22 patients, among whom there were 19 cases of pneumonia and three cases of gastroenteritis. EBV-PTLD was diagnosed in 5 patients, and all 5 patients received rituximab treatment. Hemorrhagic cystitis was also more prevalent in the reactivation group (37.5% vs. 35.2% vs. 14.1% for co-reactivation, other reactivation, and no reactivation groups, respectively, *P*=0.002) (**Table 2**).

**Table 2:** The impact of co-reactivation on clinical outcomes.

Clinical Outcomes	Co-reactivation group	Other reactivation group^#^	No reactivation group	*P* value
**neutrophil engraftment, no.(%)**	24 (100)	145 (100)	76 (97.4)	0.113
**Time of neutrophil engraftment, +d, median (range)**	13 (10–20)	13 (10–31)	14 (10–24)	0.008
**Platelet engraftment, no.(%)**	21 (87.5)	133 (91.7)	73 (93.6)	0.628
**Time of PLT engraftment, +d, median (range)**	13 (9–56)	14 (6–267)	14 (7–80)	0.389
**aGVHD, no. (%)**	12 (50)	97 (66.9)	16 (20.5)	<0.001
**Time of aGVHD, +d, median (range)**	23 (9–57)	19 (6–87)	14.5 (9–40)	<0.001
**aGVHD grade, no (%)**				0.015
** 0–II**	23 (95.8)	131 (90.3)	78 (100)	
** III–IV**	1 (4.2)	14 (9.7)	0 (0)	
**CMV viremia, no. (%)**	24 (100)	145 (100)	0 (0)	——
**Time of first CMV viremia, +d, median (range)**	33.5 (21–62)	34 (7–175)	——	——
**Duration of CMV viremia, d, median (range)**	23.5 (14–56)	18 (6–77)	——	——
**Receiving CMV-CTL**	13(54.2%)	12 (8.2%)	0	<0.001
**Highest CMV viral load, ×10^3^ copies/ml, median (range)**	28.25 (4.16–206)	9.08 (1.12–501)	——	——
**EBV viremia, no. (%)**	24 (100)	12 (8.3)	0 (0)	——
**Time of first EBV viremia, +d, median (range)**	45.5 (25–76)	58.5 (35–102)	——	——
**Duration of EBV viremia, d, median (range)**	15.5 (3–39)	14 (4–60)	——	——
**Highest EBV viral load, ×10^3^ copies/ml, median (range)**	6.75 (1.2–1760)	5.04 (0.6–536)	——	——
**Viral pneumonitis, no. (%)**	5 (20.8)	13 (9)	1 (1.3)#	0.005
**Viral enteritis, no. (%)**	0 (0)	3 (2.1)	0 (0)	0.344
**Hemorrhagic cystitis, no. (%)**	9 (37.5)	51 (35.2)	11 (14.1)	0.002
**cGVHD, no. (%)**	1 (4.2)	14 (9.7)	7 (9)	0.682
**Immune reconstitution at day 30 after HSCT, median (range)**				
** WBC, 10^9^/L**	5.24 (2.55–24.33)	5.49 (1.49–30.9)	4.73 (1.44–19.08)	0.311
** CD19, 10^9^/L**	0.0052 (0.039)	0.0034 (0.24)	0.0041 (0.042)	0.505
** CD3, 10^9^/L**	0.018 (1.66)	0.088 (7.69)	0.18 (3.02)	0.009
** CD4, 10^9^/L**	0.0031 (0.21)	0.012 (0.56)	0.072 (0.68)	<0.001
** CD8, 10^9^/L**	0.011 (1.47)	0.056 (7.28)	0.074 (2.46)	0.086
** CD4CD25, 10^9^/L**	0.00045 (0.029)	0.0017 (0.19)	0.0083 (0.27)	<0.001
**WBC count at day 60 post-transplantation, median (range)**	3.09 (0.6–7.76)	3.41 (0.65–15.77)	4.2 (0.53–9.96)	0.203
**Overall survival in 1 year after HSCT no. (%)**	12 (50)	96 (66.2)	59 (75.6)	0.021
**Leukemia free survival in 1 year after HSCT no. (%)**	12 (50)	95 (65.5)	56 (71.8)	0.057
**Mortality cause, no. (%)**				
**NRM**	9 (37.5)	27 (18.6)	12 (15.4)	0.053
**Relapse**	0 (0)	11 (7.59)	1 (1.28)	0.057
**Relapse time, d, median (range)**	——	118 (60–359)	224 (55–364)	0.262

^#^One patient who did not have CMV and EBV viremia was highly suspicious of EBV pneumonitis because of a positive EBV-DNA result in bronchoalveolar lavage fluid.

**Figure 1:**
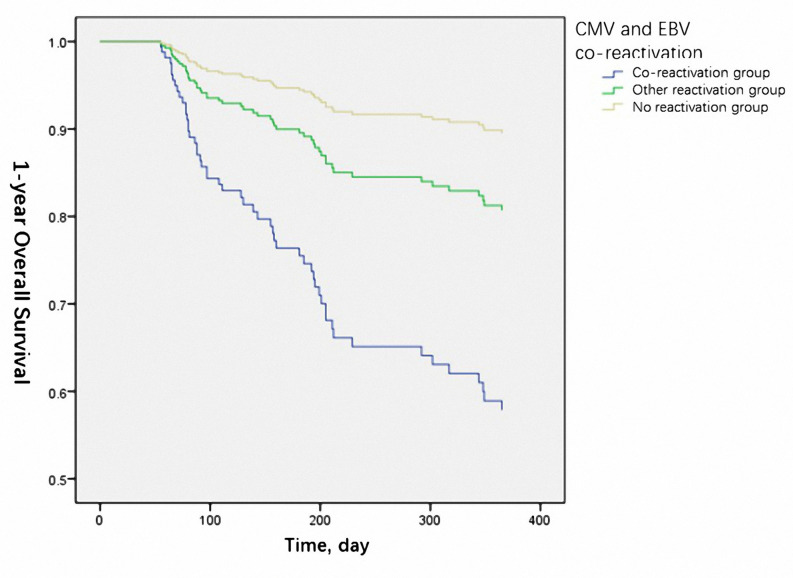
Cytomegalovirus (CMV) and Epstein-Barr virus (EBV) co-reactivation was identified as one of the independent risk factors for 1-year overall survival. (P=0.004).

**Figure 2:**
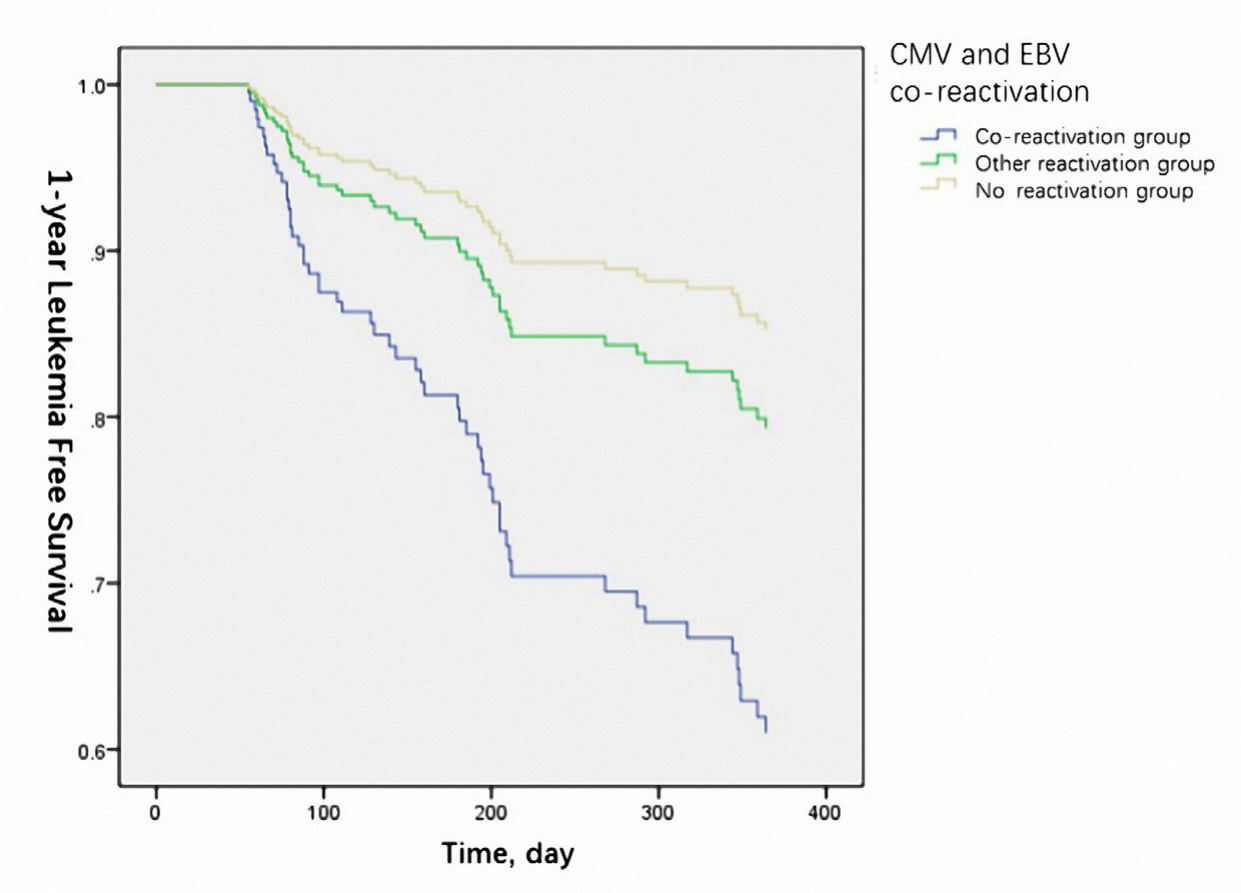
Cytomegalovirus (CMV) and Epstein-Barr virus (EBV) co-reactivation was identified as one of the independent risk factors for 1-year leukemia free survival. (P=0.016).

The 1-year OS was significantly lower in the reactivation group (50% vs. 66.2% vs.75.6% for co-reactivation, other reactivation, and no reactivation groups, respectively, *P*=0.021). The 1-year LFS was also lower in the co-reactivation group (50% vs. 65.5% vs. 71.8% for co-reactivation, other reactivation, and no reactivation groups, respectively), although the difference was not statistically significant (*P*=0.057). Viral reactivation was an independent risk factor for 1-year OS (**Figure 1**) (HR 4.94 for co-reactivation vs. no reactivation, and HR 1.94 for other reactivation vs. no reactivation, P=0.004) and LFS (**Figure 2**) (HR 3.66 for co-reactivation vs. no reactivation, and HR 1.51 for other reactivation vs. no reactivation, P=0.016). The causes of death are summarized in **Supplementary Table S1**. Risk factors for 1-year OS and 1-year LFS are summarized in **Table 3**.

**Table 3:** Risk factors for 1-year OS and 1-year LFS.

**Factors**	Univariate analysis		Multivariate analysis			
	OS	LFS	OS		LFS	
	P value	P value	P value	HR [95%CI]	P value	HR [95%CI]
**Underlying disease**	0.037(ALL vs. MDS)	0.04 (ALL vs. SAA) 0.028(ALL vs. MDS) 0.087(AML vs.MDS)	N	——	N	——
**Disease status ( CR3 or NR vs. CR1-2)**	<0.001	<0.001	<0.001	6.045 (3.088–11.832)	<0.001	5.685 (2.984–10.832)
**HLA match**	0.05 (matched sibling vs. haploidentical donor)	N	N	——	N	——
**Platelet engraftment (<=median versus >median)**	<0.001	<0.001	<0.001	0.103 (0.052–0.205)	<0.001	0.107 (0.054s–0.210)
**aGVHD grade (0–II vs. III–IV)**	<0.001	<0.001	N	——	N	——
**Virus reactivation (no reactivation vs. Co-reactivation)**	0.021	0.057	0.001	0.202 (0.078–0.527)	0.005	0.274 (0.112–0.671)
**Viral pneumonitis**	<0.001	<0.001	N	——	N	——
**Hemorrhagic cystitis**	0.002	0.002	N	——	N	——
**Highest viral load of CMV((>median versus <= median))**	0.004	0.006	N	——	N	——
**WBC count at day 60 (>median versus <= median)**	0.001	0.001	0.005	0.851 (0.734–0.988)	0.034	0.857 (0.743–0.988)

N, not statistically significant.

### Predictive Factors Associated With CMV and EBV Co-Reactivation

Patients with CMV and EBV co-reactivation were compared with all other patients to identify factors associated with co-reactivation. The donor-recipient relationship (father, mother, sibling, and son/daughter, respectively, vs. unrelated donor); HLA matched status, use of ATG; period of CMV and EBV viremia, respectively; and peak CMV and EBV DNA copies, respectively, were associated with CMV and EBV co-reactivation. CD3+ (*P*=0.052) and CD4+CD25+ (*P*=0.052) cell counts on day 30 after transplantation also seemed to play a role in virus co-reactivation in univariate analysis. Cox multivariate analysis of the above factors showed that the donor-recipient relationship (father, mother, sibling, and son/daughter, respectively, vs. unrelated donor, *P*=0.001), duration of CMV (*P*=0.014) and EBV (*P*<0.001), and CD4+CD25+ cell counts at day 30 post-transplantation (*P*=0.05) were independent risk factors for CMV and EBV co-reactivation (**Table 4**). However, of all 247 patients enrolled in the study, 45 (18.2%) patients received HSCT from HLA-matched family donors and all of these donors were siblings, which might introduce a potential bias. To account for this, we reanalyzed patients who received haplo-HSCT. The donor-recipient relationship was excluded as a risk factor for CMV and EBV co-reactivation in univariate analysis (*P*=0.561).

**Table 4:** Risk factors for cytomegalovirus (CMV) and Epstein-Barr virus (EBV) co-reactivation.

Factors	Univaraite analysis	Multivariate analysis	
	P value	P value	HR [95%CI]
**Donor-recipient relationship**	0.013 (sibling vs. father) 0.019 (sibling vs. son/daughter) 0.009 (sibling vs. unrelated matched donor)	0.001^#^ <0.001(unrelated matched donor vs. father) 0.005 (unrelated matched donor vs. sibling)	131.479(13.236-1306.056) 35.809(2.966-432.346)
**HLA match**	0.02 (identical sibling vs. unrelated matched donor)	——	——
**ATG used in conditioning therapy**	0.022	N	——
**Duration of CMV viremia (<=median versus >median)**	<0.001	0.014	1.040 (1.008-1.073)
**Duration of EBV viremia (<=median versus >median)**	<0.001	<0.001	1.155 (1.108-1.205)
**Highest viral load of CMV (<=median versus >median)**	<0.001	N	——
**Highest viral load of EBV (<=median versus >median)**	<0.001	N	——
**CD3+ cell counts at day 30 post-transplantation (<=median versus >median)**	0.052	N	——
**CD4+CD25+ cell counts at day 30 post-transplantation (<=median versus >median)**	0.052	0.05	0 (0-0.8)

^#^Donor-recipient relationship as an independent risk factor for virus co-reactivation was believed to be affected by HLA match as siblings contained all cases of HLA-identical HSCT. Reanalysis of haplo-identical HSCT patients further confirmed this hypothesis.

N, not statistically significant.

## Discussion

In this study, we demonstrated that patients with CMV and EBV co-reactivation were associated with poor prognosis in terms of acute GVHD, viral disease, OS, and LFS. This suggests that our study has important implications for clinical physicians.

Although CMV reactivation was strongly associated with EBV reactivation (13), co-reactivation of CMV and EBV was relatively less common than that of other double-stranded DNA viruses. Twenty-four (9.7%) patients were identified as having CMV and EBV co-reactivation in our study. This is consistent with a previous study in which 32/330 (9.7%) patients had co-reactivation of CMV and EBV (14), although the definition of virus co-reactivation was slightly different, as our study emphasized that the two viruses must be present at the same time. Hill et al. showed that 62% of patients could be detected with ≥2 double-stranded viruses after allogeneic HSCT. However, only 2.4% of patients were found to have CMV and EBV, with or without other double-stranded viruses (1).

Our study found that CMV and EBV co-reactivation was associated with decreased 1-year OS, which was mainly due to increased NRM. In the co-reactivation group, the 1-year NRM was higher than in the other two groups, although the difference was not statistically significant (*P*=0.053), and no death occurred because of relapse. This was partly in accordance with a previous study in which patients with CMV and EBV co-reactivation had a significant higher 6-month non-relapse mortality than those with CMV or EBV reactivation alone (14). Although CMV reactivation alone after HSCT was not associated with 1-year OS because of the decreased relapse and increased 1-year NRM (7), co-reactivation with EBV was different.

Prolonged viremia with higher CMV-load was observed in the co-reactivation group than in the other-reactivation group, reflecting the influence of parallel EBV-reactivation on CMV-replication and kinetics, which is commonly seen amongst the β-herpesviruses as they can regulate immunity. Immunoreactivation of one virus by another virus has been documented previously by us and others in both HSCT and SOT. This could also reflect the poor immune reconstitution as reflected in poor CD3+ and CD4+25+ cell counts (on day 30), which were lower than those in the other reactivation and no reactivation groups.

The incidence of acute GVHD was significantly higher in the co-reactivation and other reactivation groups than in the no reactivation group in our study. Patients in both groups had reactivated CMV, indicating an association between CMV and acute GVHD. In fact, multiple studies have shown that acute GVHD and its treatment put patients at risk of CMV reactivation (15, 16). A retrospective study also identified CMV reactivation as a risk factor for acute GVHD, proving the bidirectional relationship between CMV reactivation and acute GVHD (17). A previous study also identified grade III–IV acute GVHD as a risk factor for EBV reactivation (18). However, CMV and EBV co-reactivation in our study was not associated with a higher incidence of overall acute GVHD or severe acute GVHD (grade III-IV) than that in the other reactivation group. It might be that the other reactivation group also included patients with both reactivated CMV and EBV. However, they were not reactivated at the same period of time.

The independent risk factors for co-reactivation of CMV and EBV virus identified in this study include duration of CMV and EBV, CD4+CD25+ T cell counts on day 30 post-transplantation, and donor-recipient relationship. Reanalysis of haplo-HSCT patients was performed to account for the role of donor-recipient relationship on virus co-reactivation. As a result, the donor-recipient relationship was excluded in the univariate analysis (*P*=0.561). Previous studies have shown that risk factors for CMV reactivation after HSCT include a donor or recipient seropositive for CMV, mismatched or unrelated donors, pre-allo-HSCT viremia, and use of alemtuzumab (19, 20). However, almost all patients in our study were either donor seropositive or recipient seropositive, making it less meaningful to analyze the effect of serum status on virus reactivation.

Our study identified CD4+CD25+ cell counts on day 30 post-HSCT as an independent risk factor for CMV and EBV co-reactivation. CD4+CD25+ T cells are a subset of CD4+ T cells and represent regulatory T cells (Tregs). Normally, Tregs play an important role in controlling the cellular immune response to infectious agents, providing a balance to activating stimuli that allow elimination of the pathogen without immunopathological damage to the host. As a result, patients with a viral infection usually have an elevated number of Tregs to control the cellular immune response. However, one study showed that no significant difference could be detected by comparing both absolute and relative Treg cell numbers among allogeneic HSCT patients with and without CMV infection, indicating that Tregs did not inhibit CMV clearance in HSCT patients (21). Moreover, Ngoma et al. showed that a lower proportion of Treg on day 30 after allogeneic HSCT was associated with an increased risk of CMV infection, implying an association between impaired Treg reconstitution and CMV infection (22). The paradox might be due to the positive correlation between Treg and CMV-specific CD8+ T cell recovery after HSCT (23). Although Tregs were activated at an early stage in EBV infection (24), our study demonstrated that the effect of decreased Treg numbers on CMV reactivation was greater than that of elevated Treg numbers on EBV reactivation, as the co-reactivation group had significantly lower CD4+CD25+ cell counts.

The present study has several limitations. First, the retrospective nature of this study has inherent risks of bias; however, the patient profile and the transplant complications do not appear different from those reported in prospective studies. Second, we did not monitor other herpesviruses, which could have a bearing on all these findings. However, we concentrated only on the two most clinically important viruses with defined treatment options. We did not monitor lymphocyte reconstitution, especially virus-specific immune reconstitution, or the replication kinetics of the viruses, which could be important in managing and understanding these situations better.

Despite several limitations, we have demonstrated in this study that co-reactivation of CMV and EBV according to our definition is associated with lower 1-year OS and LFS. CD4+CD25+ T cell counts on day 30 post-transplantation are identified as one of the independent risk factors for CMV and EBV co-reactivation, which may provide an alternative way to prevent CMV and EBV reactivation in HSCT patients.

## Data Availability Statement

The raw data supporting the conclusions of this article will be made available by the authors, without undue reservation.

## Ethics Statement

The studies involving human participants were reviewed and approved by The Ethics Committee of the Peking University People’s Hospital. Written informed consent to participate in this study was provided by the participants’ legal guardian/next of kin.

## Author Contributions

X-JH and Y-QS designed the study. J-RZ, D-YS, RW and Y-QS wrote the manuscript. All authors contributed to the data preparation and interpretation. All authors approved the final version. All authors contributed to the article and approved the submitted version.

## Funding

This study was supported by the Beijing Municipal Science & Technology Commission (No. Z19110000661905), National Natural Science Foundation of China (Grant No. 81600103).

## Conflict of Interest

The authors declare that the research was conducted in the absence of any commercial or financial relationships that could be construed as a potential conflict of interest.
