# Supplementary material for: Co-Reactivation of Cytomegalovirus and Epstein-Barr Virus Was Associated With Poor Prognosis After Allogeneic Stem Cell Transplantation
Source: Front Immunol. 2021 Feb 16;11:620891. doi: 10.3389/fimmu.2020.620891 (PMC7921792; doi:10.3389/fimmu.2020.620891)
Supplement: Supplementary file 1 [file Table_1.docx]

**Supplementary table S1.** **Causes of mortality for 9 co-reactivation patients**

| Direct cause of death | no.(%) |
| --- | --- |
| Pulmonary Infection | 5(55.6) |
| Sepsis | 2(22.2) |
| Gastrointestinal hemorrhage | 1(11.1) |
| DIC | 1(11.1) |
