# Supplementary material for: Co-Reactivation of Cytomegalovirus and Epstein-Barr Virus Was Associated With Poor Prognosis After Allogeneic Stem Cell Transplantation
Source: Front Immunol. 2021 Feb 16;11:620891. doi: 10.3389/fimmu.2020.620891 (PMC7921792; doi:10.3389/fimmu.2020.620891)
Supplement: Supplementary file 2 [file Table_2.docx]

**Supplementary table S2. Risk factors for transplant-related mortality (TRM)**

| Factors | Univariate analysis | Multivariate analysis | |
| --- | --- | --- | --- |
|  | **P value** | **P value** | **HR [95%CI]** |
| aGVHD grade (0-II vs. III-IV) | ＜0.001 | **0.012** | **0.364(0.165-0.802)** |
| Disease status( CR3/NR vs. CR1/CR2) | ＜0.001 | **＜0.001** | **6.064(2.964-12.405)** |
| Virus co-reactivation versus other | 0.012 | **0.022** | **2.437(1.139-5.214)** |
| Viral pneumonitis | ＜0.001 | **0.001** | **3.716(1.734-7.965)** |
| CD4+ cell counts at day 30 post-transplantation (>=median versus <median) | 0.038 | N | —— |
| WBC count at day 60 (>=median versus <median) | 0.001 | **0.012** | **0.435(0.227-0.831)** |

*N: not statistically significant*
