# Supplementary material for: Co-Reactivation of Cytomegalovirus and Epstein-Barr Virus Was Associated With Poor Prognosis After Allogeneic Stem Cell Transplantation
Source: Front Immunol. 2021 Feb 16;11:620891. doi: 10.3389/fimmu.2020.620891 (PMC7921792; doi:10.3389/fimmu.2020.620891)
Supplement: Supplementary file 3 [file Table_3.docx]

**Table 3. Risk factors for 3-year OS and 3-year LFS**

| **Factors** | **Univariate analysis** | | **Multivariate analysis** | | | |
| --- | --- | --- | --- | --- | --- | --- |
|  | OS | LFS | OS | | LFS | |
|  | P value | P value | P value | HR [95%CI] | P value | HR [95%CI] |
| **Underlying disease** | 0.026(ALL vs. MDS)  0.015（ALL vs.SAA） | 0.026 (ALL vs. MDS)  0.015(ALL vs. SAA) | N | —— | N | —— |
| **Disease status ( CR3 or NR vs. CR1-2)** | ＜0.001 | ＜0.001 | **＜0.001** | **4.778（2.619-8.716）** | **＜0.001** | **4.644（2.548-8.465)** |
| **HLA match** | N | N | N | —— | N | —— |
| **Platelet engraftment (<=median versus >median)** | ＜0.001 | ＜0.001 | **＜0.001** | **0.087（0.048-0.157）** | **＜0.001** | **0.089（0.050-0.160）** |
| **aGVHD grade (0-II vs. III-IV)** | 0.001 | 0.001 | N | —— | N |  |
| **Virus reactivation（co-reactivation vs. no reactivation ）** | 0.040 | 0.046 | **0.019** | **2.612(1.169-5.837)** | **0.024** | **2.533（1.133-5.660）** |
| **Viral pneumonitis** | ＜0.001 | ＜0.001 | N | —— | N | —— |
| **Hemorrhagic cystitis** | 0.002 | 0.002 | N | —— | N | —— |
| **Highest viral load of CMV((>median versus <= median))** | 0.004 | 0.006 | N | —— | N | —— |
| **WBC count at day 60 (>median versus <= median)** | 0.001 | 0.001 | **0.010** | **0.525（0.322-0.856）** | **0.010** | **0.526（0.323-0.858）** |

*N: not statistically significant*
